# Acute effect of percussion and foam roller massage on flexibility, reactive and explosive strength, and muscular endurance in young adult males: a crossover pilot study

**DOI:** 10.7717/peerj.20304

**Published:** 2025-10-30

**Authors:** Peter Bartik, Martin Pacholek

**Affiliations:** Sport Sciences and Diagnostics Research Lab, Prince Sultan University, Riyadh, Saudi Arabia

**Keywords:** Percussion massage gun, Foam roller, Muscular strength, Muscular endurance, Flexibility, Performance

## Abstract

**Background:**

Percussion massage (PM) using massage guns has become popular for enhancing flexibility, muscle performance, and recovery, but evidence on its efficacy is mixed. Foam rolling (FR) is another standard modality with similar claims. This study aimed to compare the acute effects of PM and FR on hamstring flexibility, specific muscle performance types (reactive and explosive strength), and muscle fatigue.

**Methods:**

A randomized, controlled crossover design was employed with 18 physically active university students. Participants underwent three conditions: PM with Theragun, foam rolling, and a no-activation control (WA), each separated by a 4–5 day washout. Flexibility was assessed *via* the Active Knee Extension (AKE) test. Muscle performance was evaluated using a Single-Leg Reactive Strength Test (SLRST), a leg press explosive strength test, and a 30-s side hop test to assess endurance and fatigue. Statistical analyses included repeated measures ANOVA and non-parametric tests where appropriate.

**Results:**

PM significantly improved hamstring flexibility, demonstrated by lower AKE angles compared to FR and WA (*p* < 0.01), while no significant flexibility differences were found between FR and WA. No significant effects of any intervention were observed on reactive strength index, leg press power, or hop test performance. The reliability of the measurements was high (ICC > 0.84).

**Conclusions:**

PM provides an immediate enhancement in hamstring flexibility superior to FR and no treatment, but none of the interventions acutely improve muscle reactive/explosive strength or endurance. These findings suggest that PM may be an effective pre-activity tool for improving flexibility. However, it should not be relied upon as an acute performance enhancer for physically active individuals. Further research on long-term and varied protocol effects is recommended.

## Introduction

Percussion massage (PM) performed using massage guns has gained widespread popularity in recent years, particularly within sports and therapeutic contexts. These handheld devices deliver rapid, high-frequency pulses designed to deliver stimuli to deep regions of muscle tissue. Commercially available massage guns such as Hypervolt, Theragun, Ekrin, and Abox have become popular alternatives to manual physiotherapy, offering a convenient form of self-administered treatment. Their growing popularity is primarily attributed to ease of use, affordability, and claims of benefits such as increased flexibility, improved muscle performance, and enhanced recovery. Flexibility and muscular strength are key components of physical fitness frequently targeted for improvement in both athletic training and physiotherapy, as they play a critical role in determining an individual’s functional movement capacity (Özkan et al., 2024).

To date, several studies have investigated the effects of PM, though the findings remain mixed and inconclusive. Three systematic reviews have summarized the existing body of research as of 2025. A review by Martin (2021), which analyzed 39 studies, concluded that PM guns effectively increase flexibility and reduce delayed onset muscle soreness (DOMS), but do not significantly impact muscle activation or force production. In contrast, Sams et al. (2023) reviewed 13 studies and reported that PM may enhance acute and explosive muscle strength, flexibility, and reduce musculoskeletal pain. Ferreira et al. (2023), analyzing 11 studies, confirmed the positive effects of PM on flexibility but found inconsistent or even adverse outcomes regarding muscle strength, balance, acceleration, agility, and explosive performance. Based on these findings, PM guns appear effective for flexibility enhancement and pain reduction. However, the evidence regarding their effects on muscle performance remains contradictory, indicating a need for further research.

Notably, the current literature lacks detailed investigations into the impact of PM on specific types of muscular performance, particularly reactive and explosive strength, as well as muscle fatigue. Therefore, this study aims to address this gap by evaluating the acute effects of PM on these specific performance parameters.

To enhance the relevance of the findings, this study also compares PM with foam rolling (FR)—another widely used myofascial technique with similar claims. Foam rolling is commonly applied before and after exercise to improve range of motion, alleviate soreness, and potentially enhance performance. According to a systematic review (Cheatham et al., 2015), FR may improve flexibility and muscle performance in the short term, although heterogeneity in study protocols prevents firm conclusions about optimal implementation strategies. A later meta-analysis (Wiewelhove et al., 2019), based on 21 studies, suggested that FR’s effects on performance and recovery are generally minor and context-dependent. Improvements in sprint performance, flexibility, and muscle soreness reduction were observed in some cases, but the overall effect size was limited.

While both PM and FR are classified as myofascial techniques, they operate through distinct physiological mechanisms. PM delivers rapid, repetitive impulses and FR, on the other hand, applies sustained pressure and shear forces (Ferreira et al., 2023; Cheatham et al., 2015). These differing mechanisms indicate that their effects on muscle performance and flexibility may not be equivalent, thus warranting direct comparison. Given also the widespread use of both techniques, this study aimed to compare the acute effects of PM and FR on hamstring flexibility, reactive and explosive strength, and muscle endurance (fatigue). Furthermore, this represents one of the first pilot studies to directly assess and compare the effects of PM and FR on muscle fatigue. It was hypothesized that percussion massage would produce greater acute improvements in hamstring flexibility, reactive strength, explosive strength, and muscular endurance compared to foam rolling and no activation.

## Materials and Methods

### Experimental design

This study employed a single-group, within-subject controlled crossover design to evaluate various dependent variables: hamstring flexibility (degrees) using the Active Knee Extension test (AKE), contact and flight time (ms), jump height (cm) and reactive strength index (RSI) in the single leg reactive strength test, explosive strength of the lower limbs (W) during the single leg press test, and muscular endurance and fatigue (number of successful jumps) in the 30-s side hop test. The independent variables included percussion massage by Theragun (PM), foam roller (FR), and an assessment without any activation (WA). A controlled crossover design was used, where participants were randomly assigned to different treatment sequences. A standardized washout period of 4–5 days was implemented between testing sessions to minimize potential carryover effects, based on the expected duration of each intervention’s influence (Ishikawa et al., 2006). The required sample size was determined using effect sizes derived from a previous study conducted by the same research team, which followed an identical methodology (counterbalanced within-subject design, three experimental conditions, and high test–retest reliability) (Bartík & Pacholek, 2024). Using a repeated-measures analysis of variance (ANOVA) framework with a small-to-moderate expected effect size (Cohen’s f = 0.24), an alpha level of 0.05, statistical power of 0.80, and a nonsphericity correction factor (ε) of 0.75, the minimum required sample size was calculated to be 18 participants.

### Subjects

Twenty-two physically active male students from Prince Sultan University voluntarily participated in this study. Medical records and BMI data were collected to exclude individuals with injuries or those classified as overweight or obese. Additionally, students who were not physically active used PM or FL in their training routines, or regularly trained at the gym, were excluded, as these factors could influence the study results. The inclusion criteria required participants to be aged between 18 and 25 years, be recreationally physically active, defined as engaging in structured physical activity at least three times per week (Sterczala et al., 2024), and be willing to comply with the study protocol, including attending all sessions and completing assessments. Four participants did not complete the testing protocol due to health issues or other commitments, resulting in incomplete data. Ultimately, 18 students completed all measurements, and data were analyzed using a per-protocol approach. The participants had an average age of 20.17 ± 1.54 years, a height of 177.17 ± 8.90 cm, a body mass of 76.11 ± 14.14 kg, and a body mass index (BMI) of 24.11 ± 3.29. All participants were thoroughly informed about the assessment procedures and the purpose of the tests. To ensure familiarity, they were given three practice trials on each test before the first official measurement. Each participant provided electronic consent and was informed of their right to withdraw from the procedure at any time.

### Interventions

Participants were instructed to avoid physical exercise for at least 48 h and to refrain from consuming large meals at least 3 h before the intervention and measurements. All testing procedures were consistently conducted at the same time and place—the Physical Education Lab—from 12 pm to 1 pm.

Each protocol began with a standardized warm-up, which included:

1. 5 min of light intensity run on a treadmill,
2. 3 min of dynamic stretching,
3. 2 min of strength exercises (Five half squats, three lunges on each leg, and three vertical jumps using both legs).

Following the warm-up, participants either proceeded to the intervention (percussive massage by Theragun PM or foam rolling massage by using roller FR) or had a passive break in supine position of 8 min before the assessment, ensuring no additional activation. All participants completed the procedures in counterbalanced conditions.

The percussive massage was applied using the Theragun Pro model (Therabody, Los Angeles, CA, USA) for 2 min per muscle group on the jumping leg. The massage sequence was as follows:

1. Posterior calf area (m. triceps surae),
2. Posterior thigh area (m. biceps femoris, m. semimembranosus, m. semitendinosus),
3. Anterior thigh area (m. quadriceps femoris).
4. Gluteus maximus and minimus

The total duration of the percussive massage was 8 min, utilizing a percussion rate of 2,400 percussions per minute with the standard attachment head, as recommended by Theragun™. Participants lay prone on a physiotherapy table during the massage and were instructed to remain relaxed. The massage was applied by using continuous, tolerable pressure, moving from the proximal to the distal area and back. The Theragun was always held in a vertical, perpendicular position to the muscle, ensuring consistent application (Konrad et al., 2020). The protocol was administered by a single physiotherapist with 15 years of professional experience to ensure consistency.

The foam rolling massage was performed under supervision following the same protocol as the Theragun PM intervention. Participants used a foam roller to target four specific muscle groups, applying only their body weight while maintaining moderate pressure within a self-reported range of 5 to 7 on a numerical subjective scale (0 = no discomfort, 10 = maximum discomfort) (Kiyono et al., 2022). The total duration of the FR massage was 8 min. The sequence of muscle groups, massage direction, and duration were identical to the PM protocol. Participants were instructed to move slowly and with control, focusing on tender or tight areas to maximize muscle release. Participants assumed a seated position for the hamstrings, calves, and gluteal muscles, placing the foam roller beneath the targeted muscle. To apply pressure effectively, the legs were elevated for the hamstrings and calves while they remained supported for the glutes. Participants placed their hands on the ground, fingers pointing toward the body, to support a portion of their body weight. Participants adopted a prone position for the quadriceps, resting on their forearms in a plank-like stance, with the foam roller positioned under their thighs (Casado et al., 2025). The same physiotherapist administered this protocol to ensure consistency.

Following a standardized warm-up and the intervention protocols, participants completed the following tests in sequence:

(1) Active Knee Extension Test
(2) Single-Leg Reactive Strength Test
(3) Power-Based Single-Leg Press Test
(4) 30-s Side Hop Test

### Assessment methods

The AKE test (AKE) assessed hamstring flexibility by measuring active knee extension with the hip stabilized at 90° flexion. Participants, lying supine, actively extended the knee until maximal extension or compensatory movement occurred. A handheld goniometer (Baseline 12) was utilized to measure the knee angle in the sagittal plane. The fulcrum was positioned over the lateral femoral epicondyle, the stationary arm aligned with the femur, and the movement arm aligned with the fibula. Lower angles indicated greater flexibility. The test demonstrates excellent reliability, ICC = 0.99 (Gajdosik & Lusin, 1983).

The Single-Leg Reactive Strength Test (SLRST) was used to evaluate the reactive strength and power of the lower limbs. Participants began in an upright single-leg stance, performed a brief countermovement to a self-selected depth, and executed a slight forward jump, immediately followed by a maximal vertical jump, landing on the same leg. Participants were instructed to keep their hands on their hips, jump as high as possible, and minimize ground contact time. Three successful trials were recorded, with 20 s of rest between jumps. The highest jump height (cm), flight time (s), and contact time (s) were measured using the valid and reliable Optogate System (Microgate, Bolzano, Italy). The Single-Leg Reactive Strength Test using the Optogate System is likely to demonstrate high reliability (ICC > 0.85) (Glatthorn et al., 2011). The jumping limb was determined by asking participants to jump on one leg of their choice without specific instructions. To ensure consistency, participants were required to wear the same shoes for each measurement. The Reactive Strength Index (RSI) was calculated to assess participants’ ability to rapidly transition from eccentric to concentric muscle action, reflecting their efficiency in utilizing the stretch-shortening cycle. RSI was determined by dividing jump height (in meters) by ground contact time (in seconds), in accordance with the method described (Ebben & Petushek, 2010).

The power-based single-leg press test was used to assess explosive strength and power output of the lower limbs during the concentric phase. Five days prior to testing, participants completed a one-repetition maximum (1RM) assessment. After the warm-up, participants performed single repetitions with progressively increasing loads. The weight was increased by 5–10% increments until the participant could no longer complete a full repetition with proper form. Based on their results, an individualized load of 60–70% of their 1RM was assigned to their jumping leg. A designated spot (positioned centrally) on the foot platform indicated where participants should place their jumping foot, and the safety stop was adjusted individually according to each participant’s height. During the testing, participants performed three attempts to generate maximum explosive concentric contraction, starting from the safety stop position (the knee reaches approximately 90 degrees) to full knee extension. Rest intervals of 30–45 s were provided between repetitions, and the attempt with the highest power output was recorded. Power measurements were obtained using the FitroDyne Premium (FITRONIC, Bratislava, Slovakia). Research has shown that the FitroDyne provides reliable measurements for multi-joint movements (Fernandes, Lamb & Twist, 2016). The test has excellent test-retest reliability, with Intraclass Correlation Coefficients ranging from 0.94 to 0.99, and strong validity, with correlations between 0.75 and 0.88 (*p* < 0.001) (Pontiff, Li & Moreau, 2023).

The 30-s side hop test was used to assess muscular endurance and fatigue. Participants stood on their jumping legs with hands on their hips and performed side-to-side hops between two parallel lines 40 cm apart, completing as many hops as possible within 30 s. A hop was not counted if the foot landed on the line, if the non-jumping foot touched the floor, or if the hands were not holding the hips. The total number of valid hops was recorded (Kockum & Annette, 2015). Each attempt was video recorded to ensure accurate analysis of correct hops. The break between selected tests was 2–3 min. The 30-s side hop test has good intra-rater reliability, with intraclass correlation coefficients (ICC) ranging from 0.63 to 0.67, and very good inter-rater reliability, with ICCs between 0.83 and 0.91 (Kamonseki et al., 2018).

### Statistical analyses

All statistical analyses were conducted using IBM SPSS Statistics version 25. Descriptive statistics, including means and standard deviations (SD), were calculated to summarize the data. The Shapiro-Wilk test assessed the normality of all variables. For non-parametric data, the Friedman test was used to compare flexibility test results across the three related conditions (PM, FR, and WA). When significant differences were identified, pairwise comparisons were performed using Wilcoxon signed-rank tests with Bonferroni correction, applying a significance threshold of *p* ≤ 0.017. For significant *post hoc* results, Kendall’s coefficient of concordance (W) was calculated to evaluate the consistency among repeated measures. Effect sizes for pairwise comparisons were calculated using the formula $r = \displaystyle{{\left| Z \right|} \over {\sqrt N }}$, where Z is the Wilcoxon test statistic and N is the number of observations. Effect sizes were interpreted similarly to Pearson’s correlation coefficient, with thresholds of 0.1, 0.3, and 0.5 indicating small, medium, and large effects, respectively. Confidence intervals (CIs) were also calculated for all effect sizes to assess precision and reliability.

For parametric data, one-way repeated-measures ANOVA was conducted. Measurement reliability was assessed using the two-way random-effects intraclass correlation coefficient (ICC), interpreted as follows: <0.5 (poor), 0.5–0.75 (moderate), 0.75–0.9 (good), and >0.9 (excellent) (Koo & Li, 2016). To determine the practical significance of findings, effect sizes (Cohen’s d) were reported and classified according to Cohen’s guidelines as small (d = 0.2), medium (d = 0.5), and large (d = 0.8) (McLeod, 2019).

## Results

Table 1 presents the means and standard deviations for flexibility (degrees) measured by the AKE test across the three conditions: PM, FR, and WA. Statistical analysis indicated a significant overall difference in flexibility among the conditions, suggesting that at least one condition differed from the others. Measurement reliability was confirmed with a good ICC, and Kendall’s coefficient of concordance (W = 0.529, *p* < 0.001) demonstrated a moderate-to-strong level of consistency across repeated measures.

**Table 1:** Data analysis of the flexibility across different interventions (PM, FR, and WA).

	PM		FR		WA		Inferential statistics			
Fitness test	Mean	S.D	Mean	S.D	Mean	S.D	df	Chi-square	*p* value	ICC
AKE (deg.)	15.67	12.54	24.72	15.01	25.28	15.11	2	19.043	0.001	0.847

**Note:**

PM, percussion massage by Theragun; FR, foam roller; WA, without activation; SD, standard deviation; df, degrees of freedom; ICC, Intra class correlation coefficient; AKE, active knee extension test.

Pairwise comparisons revealed significant differences between PM and FR (*Z* = −3.069, *p* = 0.002) and between PM and WA (*Z* = −3.731, *p* = 0.001), while no significant difference was found between FR and WA (*Z* = −0.872, *p* = 0.383). These results indicate that the PM condition was associated with significantly lower flexibility scores compared to both FR and WA, which did not differ significantly from each other.

Effect size calculations supported these findings, with values ranging from small to very large. The comparison between PM and FR yielded a large effect size (*r* = 0.723, 95% CI [0.261–1.000]), and between PM and WA a very large effect size (*r* = 0.880, 95% CI [0.420–1.000]). The FR *vs* WA comparison showed a small effect size (*r* = 0.205, 95% CI [0.000–0.670]), consistent with the nonsignificant statistical result.

As shown in Table 2, there were no statistically significant differences between conditions across any of the fitness tests or the RSI. The observed effect sizes, reported as partial eta squared (η^2^), ranged from negligible to small to moderate, with 95% confidence intervals as follows:

**Table 2:** Data analysis of the reactive jump, leg press, and hop test across different interventions (PM, FR, and WA).

	PM		FR		WA						
Fitness tests	Mean	S.D	Mean	S.D	Mean	S.D	df	F value	*p* value	Partial ETA squared	ICC
SLRST (Tc)	427.44	89.94	426.11	83.61	416.33	80.57	2	0.817	0.45	0.046	0.96
SLRST (Tf)	411.5	49.92	409.39	54.67	411.22	54.87	1.427	0.071	0.874	0.004	0.959
SLRST (H)	20.95	5.04	20.88	5.39	21.16	5.42	1.451	0.142	0.8	0.008	0.968
Leg press (W)	672.44	152.05	651.56	187.93	655.06	216.57	1.349	0.594	0.497	0.034	0.962
Hop test (n)	41.39	12.08	39.94	12.99	37.33	13.12	2	2.611	0.088	0.133	0.926
RSI	47.89	15.68	48.21	18.31	50.19	18.25	2	1.073	0.353	0.059	0.97

**Note:**

PM, percussion massage by Theragun; FR, foam roller; WA, without activation; SD, standard deviation; df, degrees of freedom; ICC, Intraclass correlation coefficient; SLRST, Single-Leg Reactive Strength Test; Tc, contact time (ms); Tf, flight time (ms), H, Hight of the jump (cm); RSI, Reactive Strength Index.

SLRST contact time: η^2^ = 0.046, 95% CI [0.00–0.18]

SLRST flight time: η^2^ = 0.004, 95% CI [0.00–0.05]

SLRST jump height: η^2^ = 0.008, 95% CI [0.00–0.07]

Leg press power output: η^2^ = 0.034, 95% CI [0.00–0.15]

30-s side hop test: η^2^ = 0.133, 95% CI [0.00–0.31]

Reactive Strength Index (RSI): η^2^ = 0.059, 95% CI [0.00–0.20]

None of the results reached statistical significance (all *p* > 0.05). However, the 30-s side hop test demonstrated the largest effect size.

## Discussion

The primary aim of this study was to compare the acute effects of percussive massage and foam rolling on flexibility and lower limb muscle performance. This experiment examined three different interventions—PM, FR, and control assessment WA—on hamstring flexibility and physical performance, particularly reactive strength index, leg press, and 30-s side hop test. Each of the interventions lasts 8 min. The AKE test, which inversely measures flexibility (lower angles indicate greater range of motion), revealed significant differences among the conditions. Specifically, PM resulted in significantly lower AKE angles compared to both FR and WA, indicating superior immediate improvements in hamstring flexibility following percussion massage. In contrast, flexibility outcomes for FR and WA did not differ significantly, suggesting that foam rolling did not provide a meaningful short-term benefit over no intervention. The reliability analysis supported the consistency of these measurements, with a strong ICC (ICC = 0.847) and a moderate-to-strong Kendall’s coefficient of concordance (W = 0.529, *p* < 0.001), confirming the robustness of the AKE test in this context. Effect size calculations supported these findings. The comparison between PM and FR yielded a large effect size, and between PM and WA a very large effect size.

Furthermore, we evaluated muscle performance using the following tests: The Single-Leg Reactive Strength Test, which assessed lower-limb reactive strength and stretch–shortening cycle efficiency; the Power-Based Single-Leg Press Test, which evaluated maximal lower-limb power output; and the 30-s Side Hop Test, which measured dynamic balance, agility, and muscular endurance. Despite the improvements in flexibility observed with PM, no significant differences were found across the subsequent reactive strength index, leg press, or side hop test performance measures. These findings suggest that acute gains in hamstring flexibility do not necessarily translate to immediate enhancements in strength or explosive performance parameters. Furthermore, none of the interventions—PM, FR, or WA—had any measurable effect on lower-limb muscle performance, as indicated by the absence of significant differences in all three performance tests.

The improvement in flexibility observed following PM in this study aligns with findings from previous research reporting similar outcomes (Sams et al., 2023; Ferreira et al., 2023; Ahmed et al., 2024; Matsuo et al., 2025). In contrast, the lack of flexibility improvement following FR observed here diverges from the conclusions of a systematic review (Cheatham et al., 2015), which suggested that FR can produce short-term increases in joint range of motion (ROM) without impairing muscle performance. While our findings did not support a flexibility-enhancing effect of FR, they are consistent in showing no reduction in performance. Similarly, the meta-analysis (Wilke et al., 2020) concluded that FR is an effective method to acutely increase joint ROM. One possible explanation for this discrepancy is the shorter intervention duration used in our study. While the intensity of application was comparable, the 8-min FR protocol may have been insufficient to elicit the same effects reported in prior studies. Under these conditions, PM may represent a more efficient method for producing immediate flexibility gains.

Regarding muscle performance, a positive acute effect of PM has been reported in the recent systematic review (Sams et al., 2023), particularly concerning explosive strength. Another study (García-Sillero et al., 2021) similarly suggested that PM can help delay the decline in movement velocity during exercise. However, our findings did not reveal any immediate positive or negative effects of PM on reactive strength, explosive performance, or muscular endurance. These results are in agreement with the systematic review by Ferreira et al. (2023), which found no consistent effects of PM on strength, balance, acceleration, agility, or power. Conversely, a detrimental effect of PM, where participants performed fewer repetitions and sets when PM was applied during strength training was reported (Greene, Ruiz-Ramie & Craig-Jones, 2024). This outcome was not observed in our study, possibly due to differences in protocol, specifically, the use of PM immediately prior to isolated performance tests rather than during prolonged or fatiguing exercise.

The effect of FR on reactive and explosive strength, as well as muscular endurance, was not statistically significant in this study. This finding is consistent with the conclusions of the meta-analysis (Wiewelhove et al., 2019), which suggests that the effects of FR on performance and recovery are generally small and negligible.

Several limitations should be acknowledged. First, the sample size was relatively small, which may limit statistical power and the generalizability of results. Second, the study measured only short-term, acute responses, without assessing potential cumulative effects of repeated interventions. Third, the lack of long-term follow-up prevents conclusions about sustained outcomes or adaptations over time. Additionally, only a specific population was tested; thus, extrapolating results to other groups (*e.g*., older adults, females, elite athletes, injured populations) should be done with caution. Lastly, although a counterbalancing design was employed with two intervention groups and one control group, baseline measurements were not obtained.

From a practical standpoint, the results suggest that PM can serve as an effective short-duration pre-activity strategy for increasing hamstring flexibility without compromising performance. Notably, PM was superior to FR and the control condition in producing immediate flexibility gains. This may be particularly useful for physical therapists and individuals seeking to enhance mobility rapidly prior to training or competition. However, since neither PM nor FR demonstrated improvements in explosive strength and endurance performance measures, these modalities should not be considered effective acute performance enhancers.

Future studies should explore longer intervention durations, different intensities, and altering PM machines conditions (revolutions per minute, stall force, amplitude, *etc*.). Moreover, repeated-measures designs over several days or weeks could reveal potential cumulative or adaptive effects of PM and FR on performance, mobility, and recovery.

## Conclusions

Percussive massage acutely enhanced hamstring flexibility, as evidenced by a significant reduction in active knee extension angle. In contrast, foam rolling and the control condition (no intervention) produced no statistically significant changes. Despite this immediate gain in flexibility, there was no impact on subsequent performance outcomes. No significant differences were observed between interventions in reactive strength index, leg press strength, or single-leg side hop test performance. Therefore, neither percussive massage nor foam rolling had a measurable effect on muscle reactive and explosive strength and endurance.

## Supplemental Information

10.7717/peerj.20304/supp-1 Supplemental Information 1 Raw data.

10.7717/peerj.20304/supp-2 Supplemental Information 2 Consort checklist.

10.7717/peerj.20304/supp-3 Supplemental Information 3 Consort flow diagram.

## Additional Information and Declarations

### Data Availability

The following information was supplied regarding data availability:

The raw data is available in the Supplemental File.
