# Supplementary figures and images for: Acute effect of percussion and foam roller massage on flexibility, reactive and explosive strength, and muscular endurance in young adult males: a crossover pilot study
Source: PeerJ. 2025 Oct 30;13:e20304. doi: 10.7717/peerj.20304 (PMC12579856; doi:10.7717/peerj.20304)

## CONSORT Flow Diagram

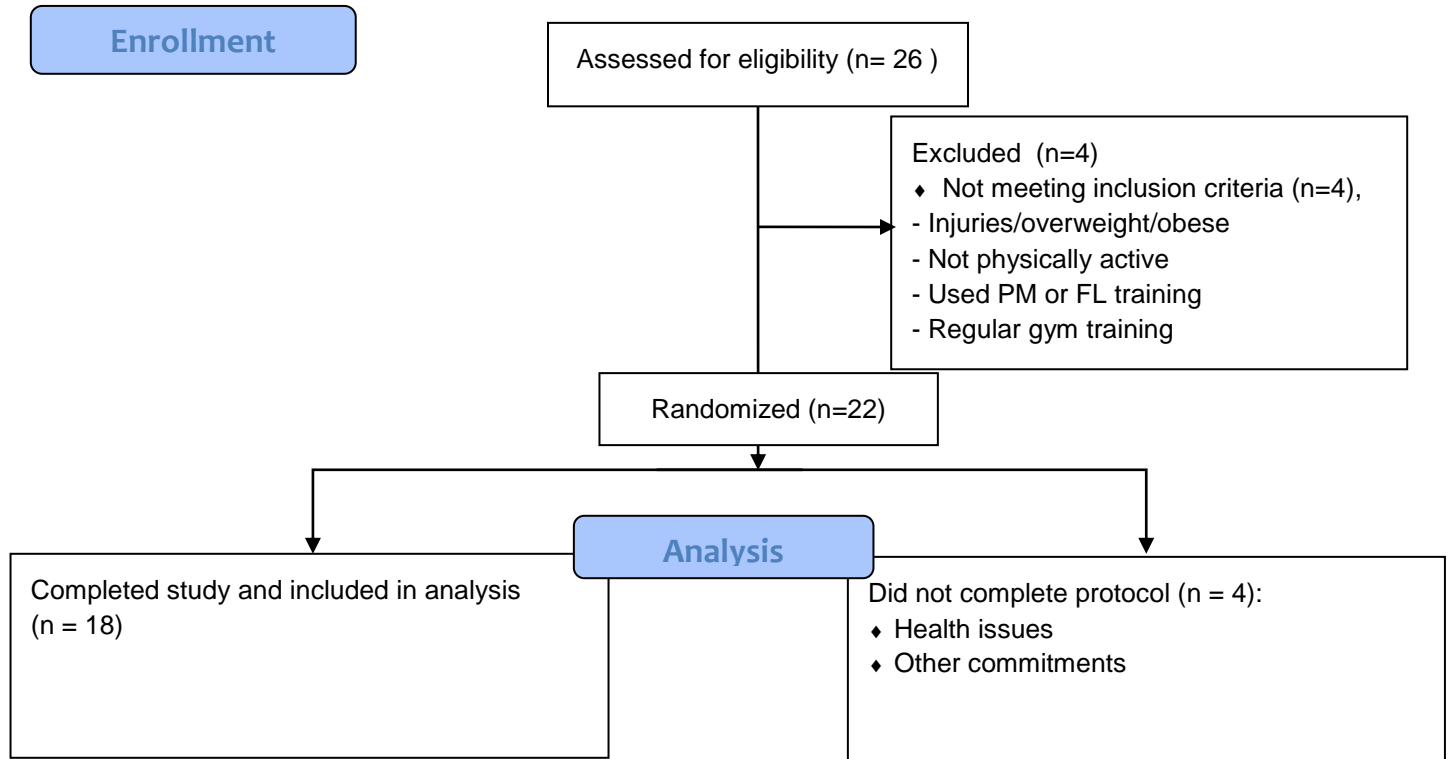

Supplement: Supplemental Information 3 [file peerj-13-20304-s003.pdf]
